# Patient-Initiated Discontinuation of Tyrosine Kinase Inhibitor for Chronic Myeloid Leukemia

**DOI:** 10.1155/2020/9571691

**Published:** 2020-03-23

**Authors:** Stephen E. Langabeer, Rehman Faryal, Michael O'Dwyer, Sorcha Ní Loingsigh

**Affiliations:** ^1^Cancer Molecular Diagnostics, St. James's Hospital, Dublin D08 W9RT, Ireland; ^2^Department of Haematology, University Hospital Galway, Galway H91 YR71, Ireland; ^3^National University of Ireland, Galway H91 TK33, Ireland

## Abstract

The introduction of tyrosine kinase inhibitors (TKI) has revolutionised the management of patients with chronic myeloid leukemia (CML) over the last twenty years, but despite significant improvements in survival, patients exhibit long-term side effects that impact on quality of life. A major advance in CML management has been the ability to discontinue TKI therapy achieving a treatment-free remission (TFR), yet this option is only available to eligible patients who present with low-risk disease and who subsequently attain deep and sustained molecular responses. A case is described of a patient with CML who self-initiated stopping of TKI therapy when in a less than optimal molecular remission. Despite this action, the patient continues to experience a TFR with prospective close molecular monitoring performed. It is emphasized that this approach may lead to ineffective treatment discontinuation, molecular relapse, and increased patient anxiety. As TFR for patients with CML moves from clinical trials into routine clinical practice, emphasis is placed on adherence to (evolving) guidelines critical to ensure optimal counselling, selection, monitoring, and continued management of patients whether TFR is successful or not.

## 1. Introduction

Chronic myeloid leukemia (CML) patients with optimal responses to tyrosine kinase inhibitors (TKI) have achieved long-term survival with life expectancy in younger CML patients approaching near normal [1]. Despite this improved outcome, long-term use of TKIs is associated with adverse events that may severely affect patient quality of life and impact on morbidity and mortality [2]. In the last decade, the remarkable phenomenon of treatment-free remission (TFR) has been witnessed: in a pivotal study, approximately 40% of CML patients on imatinib therapy for more than two years and in a deep molecular response remained in sustained clinical and molecular remission upon antileukemic TKI discontinuation [3]. TFR rates of 40–60% in eligible patients have been corroborated by numerous, subsequent, randomised clinical trials (in which the eligibility criteria of TKI, time on TKI, and length and depth of molecular response have varied) and have been recently reviewed [4, 5]. Outside of clinical trials, similar TFR rates are also achievable in the “real-world” setting [6–8]. Importantly, in all these studies where patients relapse after discontinuation (nearly always within the first six months of stopping), reintroduction of a TKI results in attainment of a favourable molecular response in the vast majority of patients [9]. The persistence of quiescent CML stem cells in those patients in successful TFR suggests some form of immunological interaction is partly responsible for control of the residual leukaemic clone, the mechanisms of which remain poorly defined [10, 11]. Of note is the recurrent adverse event in 20–30% of those CML patients attempting TFR of a transient TKI withdrawal syndrome manifesting as musculoskeletal pain [12].

With the increased acceptance and uptake of attempting TFR in routine clinical practice, recommendations for the minimal requirements for treatment discontinuation have been proposed by both European and North American experts groups [13, 14]. Similarities exist between these two sets of criteria although there remains limited consensus on the requirements for TKI treatment duration or depth and stability of the molecular remission prior to attempting TFR [15]. Both sets of guidelines concur on the importance of instigating frequent molecular monitoring so that molecular relapse can be rapidly captured prompting reintroduction of TKI.

Improving quality of life may alone provide sufficient rationale for TFR consideration. Younger patients may have a desire to lessen the potential of future adverse events or by personal/family goals, whereas older patients may seek to mitigate the adverse effects they currently experience on TKI therapy [16]. Given that nonadherence is not an uncommon pattern in patients on long-term TKI therapy [17] and an increased awareness of TFR, CML patients may be independently motivated to stop therapy.

## 2. Case Report

A 55-year-old man presented in November 2008 with fatigue, headache, left upper quadrant abdominal discomfort, and palpable splenomegaly. He had a hemoglobin of 11.6 g/dL, a white cell count of 53.7 × 10^9^/L, and platelets of 165 × 10^9^/L. Bone marrow aspirate revealed moderate hypercellularity with less than 2% myeloblasts, and cytogenetics demonstrated a karyotype of 46,XY,t(9;22)(q34;q11.2). Molecular analysis revealed high levels of e14a2 *BCR-ABL1* transcripts, all consistent with a diagnosis of chronic phase CML with a low-risk Sokal score of 0.75.

**Figure 1:**
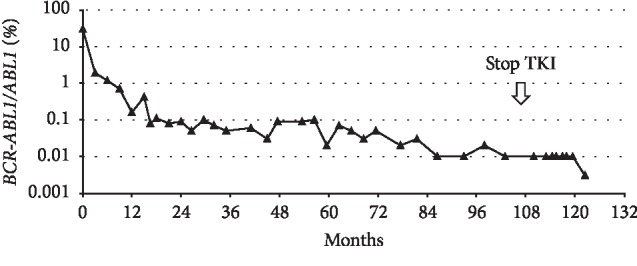
Patient *BCR-ABL1* levels throughout disease course.

The patient was enrolled on an open label, single stage, multicentre, nonrandomized, phase II clinical trial to assess the efficacy of upfront nilotinib 300 mg twice daily [18]. Prospective molecular *BCR-ABL1* monitoring was performed in a European Treatment and Outcome Study (EUTOS)-certified laboratory according to standardized procedures with results reported in line with standardized definitions of response [19, 20]. The patient achieved a major molecular response (MMR; *BCR-ABL1*/*ABL1* IS ≤ 0.1% on the International Scale) at 16 months that was maintained for seven years (Figure 1). Thereafter, a deeper molecular response (MR4; *BCR-ABL1*/*ABL1* IS ≤ 0.01% on the International Scale) was transiently noted.

During his treatment he continued to have mild fatigue and headaches. Transient grade-II increase in serum lipase was also noted which normalised on temporary interruption of nilotinib. Later in the treatment, he reported having frequent nightmares, sleep disturbances, poor concentration, and in general, poor quality of life. Overall, his treatment was continuous with three short (<7 days) interruptions due to impairment in baseline renal functions and transient increase in serum lipase at one instance but was not considered a candidate for attempting TFR at any time due to the lack of a prolonged, deep molecular response. However, at a follow-up appointment, the patient stated that he had stopped taking his TKI in March 2018 and reported feeling much better having stopped nilotinib and did not wish to restart. He had not noticed any symptoms suggestive of a TKI withdrawal syndrome. Counselled that his response could be lost and being offered treatment with an alternative TKI, he preferred to pursue a trial of treatment cessation, prompting immediate monthly *BCR-ABL1* monitoring to detect any loss of molecular response as per European guidelines [13]. The patient is now 18 months after TKI discontinuation maintaining a stable *BCR-ABL1* level of 0.01% (Figure 1) and remains clinically well. Given the less than optimal *BCR-ABL1* history, molecular monitoring continues at six weekly intervals.

## 3. Discussion

For many CML patients who have achieved stable and deep molecular responses with TKI therapy, evidence from both clinical trials and real-world settings has demonstrated the feasibility of TFR with current guidelines outlining minimal criteria for eligibility [13, 14]. While these guidelines concur that CML patients attempting TFR must be under the care of CML specialist physicians, have low-risk, chronic phase disease, and have been on continuous TKI therapy for a specified number of years, several aspects of these evolving guidelines remain equivocal (Table 1).

Initial TKI choice appears to have little impact on attaining TFR which has been achieved at a similar frequency of CML patients treated with first-line with imatinib, dasatinib, or nilotinib. The use of more potent inhibitors first-line may increase the number of patients eligible for TFR consideration and reduce overall TKI exposure [21]. Furthermore, in those patients who have switched TKI due to intolerance, TFR rates are comparable to those patients who received only a single agent, provided criteria regarding molecular response and other features are met and are superior to those patients previously experiencing resistance to their first-line TKI [16]. A further intriguing development is the de-escalation of TKI prior to cessation, recently demonstrated to improve the success of TFR protocols though the mechanism of this benefit is not yet clear [22].

Interest has focussed on the influence of the *BCR-ABL1* transcript type on response to first-line TKI with the patients expressing e14a2 transcripts having superior molecular responses to those harboring e13a2 transcripts; however, the impact on overall survival remains unclear [23, 24]. This theory has been extrapolated to those patients attempting TFR with one recent study suggesting that the e14a2 *BCR-ABL1* transcript, as expressed in the above case, favourably impacts on sustained TFR upon TKI discontinuation [25].

The initial molecular response to TKI in this case was slow and would be considered as a warning under current European LeukemiaNet guidelines, yet this did not prevent the patient achieving a subsequent TFR [26]. It is known that early molecular response and female sex strongly predict stable undetectable *BCR-ABL1* transcripts (the criteria for TKI discontinuation) [27, 28] but whether this actually translates to maintaining TFR upon cessation is not yet apparent. Most TKI discontinuation trials intuitively conclude that both the depth and duration of molecular response are key indicators of successful TFR [5] although a sustained MR4 prior to TKI cessation was not apparent in this case. While rare instances of TFR have been recently described in CML patients in only MMR [29], TFR trials to date have required deep molecular responses of a minimum of MR4 for entry. For those patients not achieving MR4, the possibility exists of clinical trials that facilitate switching to an alternative TKI in order to improve and sustain the molecular response before TFR. It is in patients with significant long-term side effects that such an approach may be beneficial.

Many factors contribute to the decision to attempt TFR in patients who are eligible and include risk of relapse, side effects, financial considerations, polypharmacy, and willingness to change something that is already working [30]. In an analysis of real-world experience of unplanned TKI discontinuation, patient request was one of the most frequent reasons for stopping with information on TFR clinical trials increasingly available through multiple media sources [31]. Acknowledging patient preference, TKI discontinuation should always follow a full assessment and consultation with patient-initiated discontinuation firmly discouraged.

Given the expected increased prevalence of CML in the forthcoming years driven by both population aging and a relative survival improvement, TFR is likely to become an increasingly sought long-term option and goal [32]. From a clinical practice perspective, an up-to-date survey of oncologists and hematologists regarding TKI therapy discontinuation practice including molecular monitoring, adequate response for discontinuation, and relapse and symptoms following discontinuation suggested discontinuation was often attempted under suboptimal conditions underscoring the requirement for clinician education [33]. Given the heterogeneity of inclusion criteria in previous TFR studies, further clinical trials, including biological investigations, are warranted to establish the optimal preconditions for achieving TFR [5]. Adherence to existing guidelines is recommended though these are likely to evolve with refinement and harmonization of TKI discontinuation trials.

## Figures and Tables

**Table 1:** Minimum criteria for tyrosine kinase inhibitor discontinuation outside of a clinical trial.

Criteria for TKI cessation	ESMO guidelines [18]	NCCN guidelines [19]
Consultation	CML speciality centre	CML speciality centre
Age	Not specified	≥18 years
Risk category or phase at diagnosis	Non-high Sokal score	Chronic phase
On TKI therapy	>5 years	>3 years
*BCR-ABL1* transcript	e13a2, e14a2 or other transcript quantifiable over 4.5 log range	Evidence of quantifiable transcript
Molecular response achieved & duration	MR4.5 achieved/MR4.0 ≥ 2 years	MR4.0 ≥ 2 years
*BCR-ABL1* test sensitivity/turnaround	At least MR4.5/4 weeks	At least MR4.5/2 weeks
Monitoring frequency	Monthly for 6 months/6 weekly for next 6 months/3 monthly thereafter	Monthly for one year/6 weekly for second year/3 monthly thereafter
TKI resumption	Not specified	Within 4 weeks of loss of MMR with monthly monitoring

TKI: tyrosine kinase inhibitor; ESMO: European Society for Medical Oncology; NCCN: National Comprehensive Cancer Network; CML: chronic myeloid leukemia; MR: molecular response; MMR: major molecular response.
